# Cuprotosis Programmed-Cell-Death-Related lncRNA Signature Predicts Prognosis and Immune Landscape in PAAD Patients

**DOI:** 10.3390/cells11213436

**Published:** 2022-10-31

**Authors:** Hao Chi, Gaoge Peng, Rui Wang, Fengyi Yang, Xixi Xie, Jinhao Zhang, Ke Xu, Tao Gu, Xiaoli Yang, Gang Tian

**Affiliations:** 1 Clinical Medical College, Southwest Medical University, Luzhou 646000, China; 2 Department of General Surgery (Hepatobiliary Surgery), The Affiliated Hospital of Southwest Medical University, Luzhou 646000, China; 3 Nuclear Medicine and Molecular Imaging Key Laboratory of Sichuan Province, Luzhou 646000, China; 4 Academician (Expert) Workstation of Sichuan Province, Luzhou 646000, China; 5 School of Stomatology, Southwest Medical University, Luzhou 646000, China; 6 Department of Laboratory Medicine, The Affiliated Hospital of Southwest Medical University, Luzhou 646000, China

**Keywords:** cuprotosis, PAAD, programmed cell death, tumor microenvironment, immunotherapy, prognostic signature

## Abstract

In terms of mortality and survival, pancreatic cancer is one of the worst malignancies. Known as a unique type of programmed cell death, cuprotosis contributes to tumor cell growth, angiogenesis, and metastasis. Cuprotosis programmed-cell-death-related lncRNAs (CRLs) have been linked to PAAD, although their functions in the tumor microenvironment and prognosis are not well understood. This study included data from the TCGA-PAAD cohort. Random sampling of PAAD data was conducted, splitting the data into two groups for use as a training set and test set (7:3). We searched for differentially expressed genes that were substantially linked to prognosis using univariate Cox and Lasso regression analysis. Through the use of multivariate Cox proportional risk regression, a risk-rating system for prognosis was developed. Correlations between the CRL signature and clinicopathological characteristics, tumor microenvironment, immunotherapy response, and chemotherapy sensitivity were further evaluated. Lastly, qRT-PCR was used to compare CRL expression in healthy tissues to that in tumors. Some CRLs are thought to have strong correlations with PAAD outcomes. These CRLs include AC005332.6, LINC02041, LINC00857, and AL117382.1. The CRL-based signature construction exhibited outstanding predictive performance and offers a fresh approach to evaluating pre-immune effectiveness, paving the way for future studies in precision immuno-oncology.

## 1. Introduction

There are now 331,000 fatalities each year caused by pancreatic cancer, a malignant tumor with a dismal prognosis and a growing global prevalence [1,2,3]. Surgical intervention is the only way to improve the chances of long-term survival [4]; however, standard radiation and chemotherapy do not appreciably extend the life of PAAD patients [5], and around 80–85% of PAAD patients develop extensive metastases that cannot be surgically removed [2]. In addition, it takes around 6 months before PAAD is diagnosed [6] because it usually lacks significant symptoms in the early stages. Different clinical features and treatment outcomes might occur in patients with the same clinical stage, suggesting that the prognosis of tumors based on traditional clinical indicators is not entirely accurate. Traditional prognostic indicators include the TNM stage, pathological grade, and histological grade [7]. Patients’ clinical stages determine the chemotherapy and immunotherapy strategies. Because of this, it is crucial to identify novel prognostic indicators for PAAD patients in order to enhance their quality of life.

In all organisms, copper is an essential cofactor in the maintenance of their life activities through mitochondrial respiration, iron uptake, and antioxidant/detoxification [8]. There are many mechanisms for cell death, but cuprotosis is different from autophagy, apoptosis, necrosis, and ferroptosis. Cuprotosis is induced by the binding of copper ions to lipoic acylated proteins. Copper ions cause a proteotoxic stress response, which ultimately kills cells by decreasing the amounts of Fe-S cluster proteins [9]. Drugs that transport copper ions, such as elesclomol, disulfiram, and NSC319726, may also be lethal to cells. The buildup of copper is closely linked to tumorigenesis, angiogenesis, and metastasis [8,10]. Currently, the findings of several studies indicated that a significant difference was detected between normal and tumor subjects in copper levels in serum and tumor tissues [11,12]. In pancreatic cancer, there is also some evidence that high levels of copper may affect the development of tumors [13] and that copper levels are significantly elevated in PAAD patients and are strongly associated with poor prognosis [14]. However, research on the mechanisms of cuprotosis regulation in tumors is still in the preliminary stage.

Long RNAs having more than 200 bases, but no protein-coding potential are known as long noncoding RNAs (lncRNAs) [15]. Epigenetic regulation, dose compensation, cell differentiation regulation, and cell cycle regulation are just a few of the many biological processes that long noncoding RNAs control [16]. Research has shown that lncRNAs are critical for progression, invasion, metastasis, and treatment resistance [17,18]. Further, numerous lncRNAs have been found to function as non-invasive indicators for the diagnosis, prognosis, and detection of PAAD [19,20,21]. The role of CRLs in PAAD prognosis and the tumor microenvironment is, however, still up in the air. To better anticipate the prognosis of PAAD patients and evaluate the efficiency of pre-treatment, investigating lncRNAs linked to copper mortality may provide a fresh viewpoint with substantial ramifications.

In our study, we analyzed the association between CRLs and immunotherapy, the immune microenvironment, and chemotherapy sensitivity and screened five CRLs based on the TCGA-PAAD cohort to create a predictive signature. Finally, qRT-PCR was used in the PANC-1 and SW1990 cell lines to investigate the function of the five CRLs in cuprotosis. Through a thorough examination of genetic data, we aimed to show that CRLs are useful for determining the prognosis of PAAD patients and can provide new tools to enhance the therapy choices.

## 2. Materials and Methods

### 2.1. Patient Data Sources

Follow-up data including TNM stage, age, sex, and overall survival were included in the TCGA-PAAD (http://cancergenome.nih.gov/) (accessed on 2 August 2022) gene expression profiles and clinical data for 178 PAAD cases. The sample size of PAAD patients with stage M was highly variable and excluded from the analysis. PAAD patients were randomized into training and test groups in a 7:3 ratio, along with genes associated with cuprotosis. The GENCODE website (https://www.gencodegenes.org/) (accessed on 2 August 2022) was used to annotate lncRNAs.

### 2.2. Model Construction and Validation

The “limma” R package differential analysis between normal and tumor groups utilized an adjusted *p*-value of 0.05 and a screening threshold of |logFC| > 1 [22]. We performed univariate Cox regression and Lasso regression using “glmnet” in R to uncover survival genes. Tenfold cross-validation was determined. The multivariate Cox regression model identified the key gene and its coefficient. We built a risk profile using five CRLs and the optimum values and accompanying coefficients. The CRL risk score was determined as follows for each individual patient: Risk level = Expression_lncRNA1_ × Coef_lncRNA1_ + Expression_lncRNA2_ × Coef_lncRNA2_ + Expression_lncRNAn_ × Coef_lncRNAn_.

### 2.3. Consensus Clustering Analysis

To analyze the CRL signature in PAAD, we used the “ConsensusClusterPlus” R software to group all PAAD patients into distinct clusters. We used the “pheatmap” R software to visualize the differential expression and clinicopathological characteristics of CRLs across clusters. The MSigDB database was used to conduct the GSVA analysis using the “c2.cp.kegg.v7.4.symbols.gmt” package. Differences in pathways across clusters were analyzed using the “GSVA” R package.

### 2.4. Model Formulae

To calculate the median values and classify all PAAD patients as high-risk, we used the R package “survminer” in conjunction with the output model equations. In order to determine the C-index, the R package “pec” was utilized. The ability to anticipate 5 CRLs was evaluated using a time-dependent ROC curve analysis.

### 2.5. Independent Prognostic Analysis and Nomogram Construction

We used univariate and multivariate Cox regression to determine whether the risk score was an independent prognostic factor. Risk scores vs. clinicopathologic variables were used to build histograms in rms in R to predict survival at 1, 3, and 5 years for PAAD patients in the TCGA cohort.

### 2.6. Functional Enrichment Analysis

Functional enrichment analysis of differentially expressed genes connected to five CRLs in PAAD was used to investigate functional annotation and enrichment pathways. ClusterProfiler was used to evaluate KEGG pathways and Gene Ontology (GO).

### 2.7. Immunity Analysis of the Risk Signature

Immune infiltration scores were measured using standard techniques such as XCELL [23,24], TIMER [25,26], QUANTISEQ [25,26], MCPCOUNT [27], EPIC [28], CIBERSORT [25,29], and CIBERSORT-ABS [30]. Immune cells and risk assessments were investigated using Spearman correlation analysis. PAAD patients were classified by immune cell features using Cibersort. Using Auslander’s data [31], we created a list of 20 potential therapeutically suppressive immunological checkpoints to compare between high-risk and low-risk groups.

Mariathasan’s research features [32] and Xu et al.’s cancer and immunity website (http://biocc.hrbmu.edu.cn/TIP/) (accessed on 2 August 2022) [33] provided a list of genes positively connected with the anti-PD-L1 drug response [27]. Gene characteristics favorably associated with cancer immunity cycles and therapy were enriched in the low-risk and high-risk groups using the GSVA algorithm [34]. The “ggcor” R package linked risk scores with those two genetic characteristics.

### 2.8. Drug Sensitivity

Based on their treatment response, PAAD patients were classified as high-risk or low-risk by their half-maximal inhibitory concentration (IC50) based on the Genomics of Drug Sensitivity in Cancer (GDSC) database (https://www.cancerrxgene.org/) (accessed on 2 August 2022) [35]. pRRophetic in R was used.

### 2.9. Somatic Mutation Analysis

The TCGA-PAAD mutation database (https://portal.gdc.cancer.gov/) (accessed on 2 August 2022) was accessed. Mutation information from PAAD samples was recorded in a mutation annotation format and evaluated with the help of the Maftools program [36]. In order to better understand the risk of PAAD, we compared patients with and without a tumor mutation burden (TMB) score. The TMB score is determined by multiplying the ratio of mutations to covered bases ×10^6^ [37].

### 2.10. Construction of Cuprotosis Cell Model and qPCR Assay

We obtained our hTERT-HPNE cells from the BeNa Culture Collection (Henan, China), and they were from a human pancreatic ductal epithelial cell line. The PANC-1 and SW1990 cell lines were acquired from the Experimental Medicine Center at Southwest Medical University, both of which are specific to the study of human pancreatic cancer (Luzhou, China). The hTERT-HPNE cells were grown in a humidified 5% CO_2_ environment in RPMI media supplemented with 10% fetal bovine serum (FBS). Both the PANC-1 and SW1990 cells were grown in a 5% CO2 and 37 °C incubator in DMEM containing 10% FBS. In agreement with the published literature [9], a 2 h pulse treatment with elesclomol-2-CuCl_2_ (100 nM elesclomol + 1 M CuCl_2_) caused cuprotosis in PANC-1 and SW1990 cells. After 24 h, total RNA was extracted using TRIzol reagent (TIANGEN, Beijing, China). Before and after the drug treatment, qPCR was used to identify changes in cuprotosis-related lncRNA expression in the hTERT-HPNE, PANC-1, and SW1990 cells. Elesclomol was purchased from Solarbio(Solarbio, Beijing, China). CuCl_2_ was purchased from Macklin Inc (Macklin, Shanghai, China). Unless otherwise stated, all reagents were obtained from Gibco (Gibco, Grand Island, NY, USA). The qPCR test primer sequences are listed in Supplementary Table S1.

### 2.11. Statistical Analysis

R was used for all bioinformatics analyses (version 4.1.0). Using the Wilcoxon rank-sum test, we found that the difference function between the two groups was statistically significant (*p* < 0.05).

## 3. Result

### 3.1. Identification of Candidate Cuprotosis-Related lncRNAs

**Figure 1:**
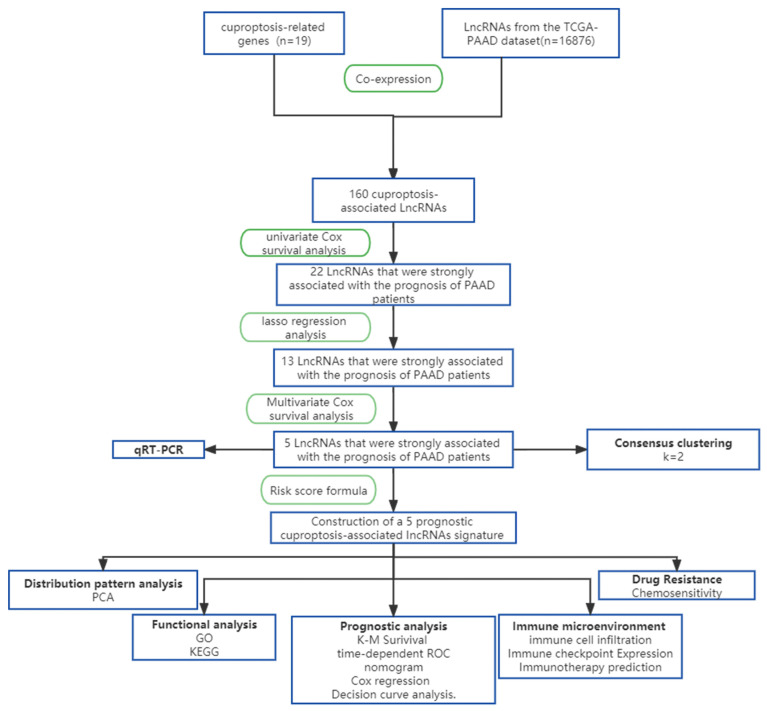
The graphic flowchart summarizes the main design of the present study.

**Figure 2:**
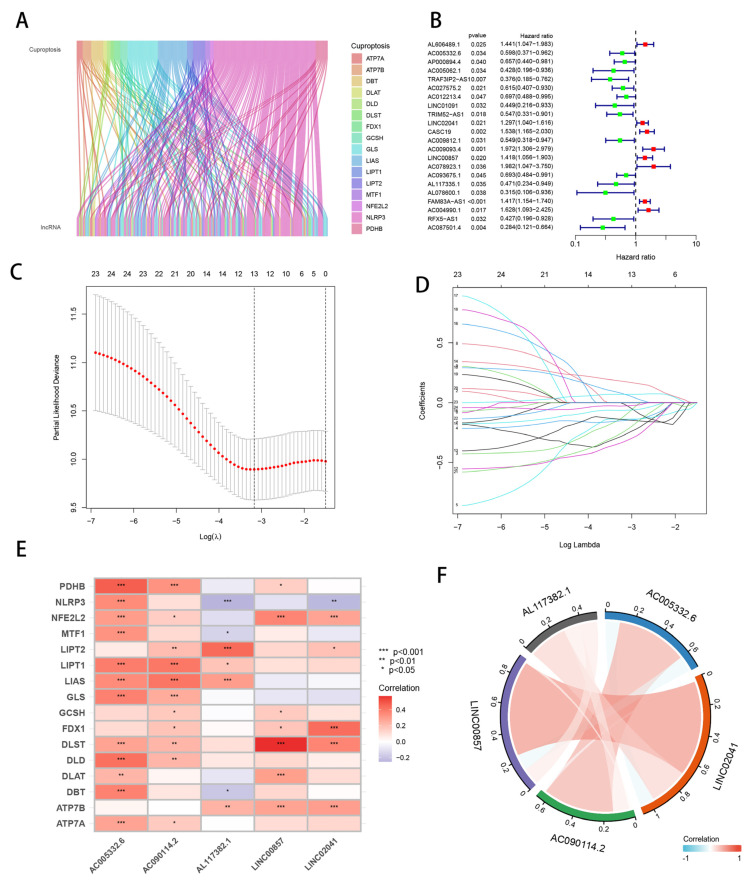
Candidate cuprotosis-related lncRNA identification. (**A**) Sankey plots illustrating the co-expression of 207 long noncoding RNAs associated with cuprotosis and the associated genes (lncRNAs). (**B**) Univariate Cox regression analysis was utilized to assess the prognosis of lncRNAs associated with cuprotosis. (**C**) Adjusted parameter selection in the Lasso model with tenfold cross-validation. (**D**) Lasso coefficient curves. (**E**) Correlation between the five lncRNAs that were evaluated and genes relevant to cuprotosis. (**F**) Correlation study of the five lncRNAs associated with cuprotosis. * *p* < 0.05; ** *p* < 0.01; *** *p* < 0.001.

The diagrammatic flowchart presents the basic framework for this investigation (Figure 1). After filtering the TCGA-PAAD dataset for protein-coding genes, we were left with 16,876 lncRNAs. It has been shown that 19 genes are involved in cuprotosis [9]. These 19 cuprotosis-related genes were used in co-expression studies with a total of 16,876 extracted lncRNAs to find cuprotosis-related lncRNAs (Figure 2A). Once we had found 160 lncRNAs related to cuprotosis, as shown in Figure 2B, we immediately subjected them to univariate Cox analysis. Following a search for lncRNAs with significant associations with PAAD patient outcomes, 22 cuprotosis-associated lncRNAs were subjected to Lasso regression analysis. From this, 13 lncRNAs were isolated, and their regression coefficients and cross-validation trends were analyzed (Figure 2C,D). Finally, these high-dimensional data were downscaled by a multifactorial Cox proportional risk regression model, and five CRLs were finally identified, namely, AC005332.6, LINC02041, AC090114.2, LINC00857, and AL117382.1. The corresponding regression coefficients were also obtained, which were −0.6849, 0.2587, −0.4467, 0.3234, and −0.6357. In the multivariate Cox analysis, the linear prediction model was built based on the five CRLs weighted by their regression coefficients. The five CRLs were weighted by their correlation coefficients with the formula: risk score = (−0.6849 × AC005332.6 expression level) + (0.2587 × LINC02041 expression level) + (−0.4467 × AC090114.2 expression level) + (0.3234 × LINC00857 expression level) + (−0.6357 × AL117382.1 expression level). Further analysis revealed a strong association between cuprotosis-related genes and CRLs (Figure 2E), while the five CRLs were significantly correlated with each other (Figure 2F).

### 3.2. Consensus Clustering Identified the Molecular Subtypes of PAAD

**Figure 3:**
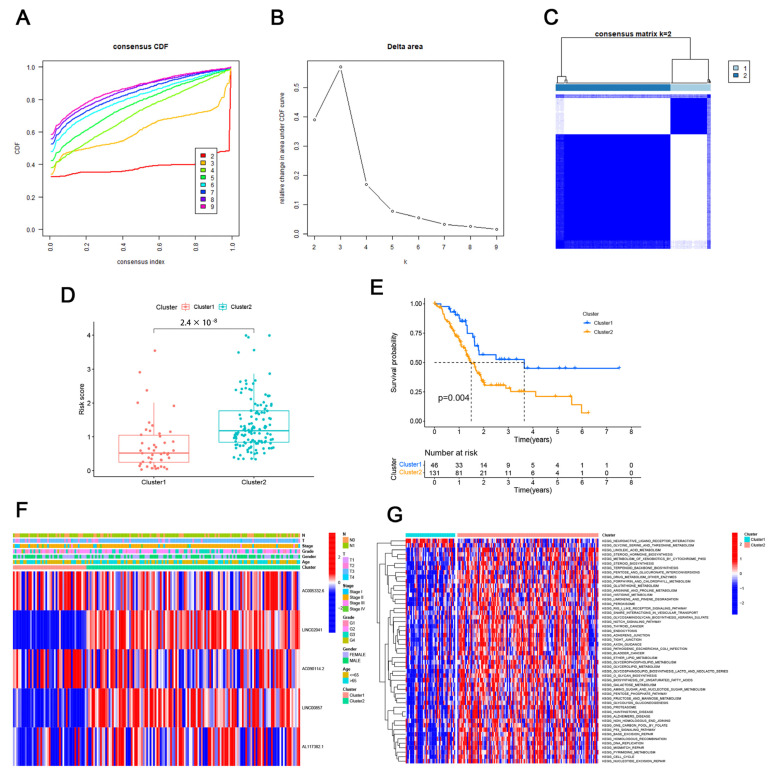
The molecular subgroups of PAAD were discovered using consensus clustering. (**A**) Cumulative distribution function (CDF) for consensus clustering for k = 2 to 9. (**B**) Relative variance in the area of the cumulative CDF curve for k = 2 to 9. (**C**) Consensus matrix with k = 2. (**D**) The difference in risk scores between Clusters 1 and 2. (**E**) The OS differences between Clusters 1 and 2. (**F**) Clinicopathological factors and connections between CRL expressions. (**G**) Examining the KEGG enrichment of several clusters.

The “ConsensionClusterPlus” R program was used to conduct the consensus clustering study. We selected k = 2 as the best aggregation stability in the consensus clustering based on the expression of cuprotosis-related lncRNAs (Figure 3A–C). According to Figure 3D, Cluster 1 had significantly higher risk scores than Cluster 2, indicating that Cluster 2 had a higher concentration of high-risk scores (*p* = 2.4 × 10^−8^). Using the ClusterSurvival R program, we found that some patients’ OS was significantly better in Cluster 1 than in Cluster 2 (*p* = 0.004) (Figure 3E). Figure 3F shows a heat map of the connection between the five CRLs’ expression and clinicopathological characteristic parameters. The two groups showed notable differences in CRL expression. To elucidate the underlying biological pathways, we performed enrichment analysis of different cluster samples using KEGG enrichment analysis and found associations with various cancer-related pathways such as the cell cycle, bladder cancer, thyroid cancer, and base excision repair (Figure 3G).

### 3.3. Validation of CRL Signature and its Prognostic Value

**Figure 4:**
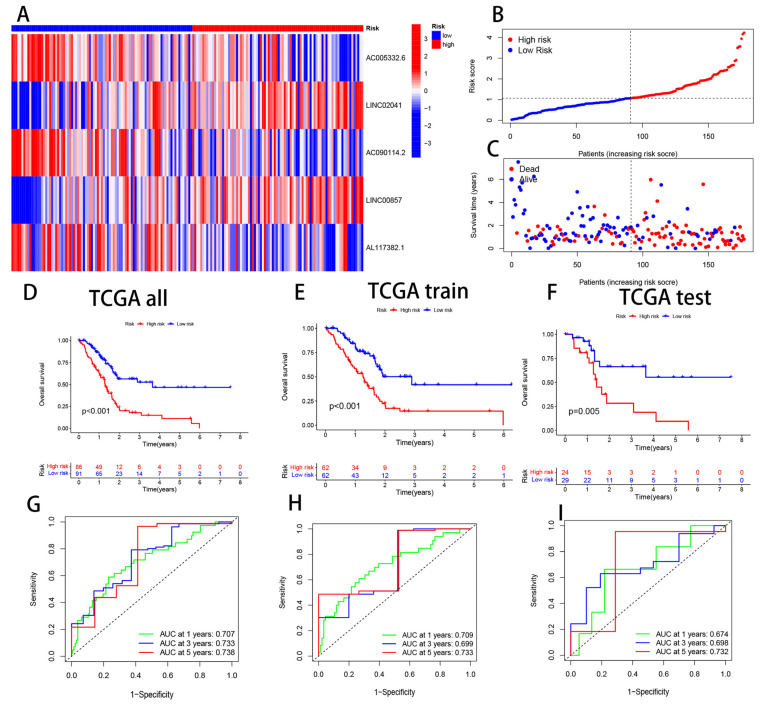
Validation of the accuracy of the CRL models to predict patient prognosis. (**A**) A heat map showing the differential expression of five CRLs in groups at high and low risk. (**B**,**C**) Scatter plots of PAAD patients’ risk ratings and prognostic cuprotosis-related lncRNAs that are connected with survival time. (**D**) All TCGA cohorts’ survival curves. Survival curves for the TCGA training and test cohorts are shown in (**E**,**F**), respectively. The TCGA patient cohorts with time-dependent ROC curves are shown in (**G**) for all patient cohorts and (**H**) for the training cohort. (**I**) TCGA test cohort time-dependent ROC curves.

The five CRL models were validated by stratifying PAAD patients into high-risk and low-risk groups based on the median cut-off point of their prognostic risk scores. We initially compared the expression levels of all five CRLs across the high-risk and low-risk groups. AC005332.6, AC090114.2, and AL117382.1 were considerably higher in the low-risk group, whereas LINC02041 and LINC00857 were lower (Figure 4A). The methodology applied to the complete dataset showed that PAAD patients’ mortality rate increased with the risk score (Figure 4B,C). In the encore cohort (*n* = 177), high-risk patients obtained worse outcomes than low-risk individuals (*p* < 0.001) (Figure 4D). The risk score’s 1-, 3-, and 5-year OS prediction AUC values of 0.707, 0.733, and 0.738 demonstrate the prediction model’s high specificity and sensitivity (Figure 4G). To test our prognostic model, we randomly split the study population into a training cohort (*n* = 124) and a test cohort (*n* = 53) with a training/test ratio of 7:3. High-risk training patients experienced worse clinical outcomes than low-risk patients (Figure 4E). In the time-dependent ROC curve, the 1-year AUC was 0.709, the 3-year AUC was 0.699, and the 5-year AUC was 0.733 (Figure 4H). We identified the same trends in the test cohort. High-risk patients fared worse than low-risk patients (*p* = 0.005) (Figure 4F). The time-dependent ROC curve showed AUC values of 0.674, 0.698, and 0.732 at 1, 3, and 5 years (Figure 4I). Our five-CRL predictive signature performed well based on these data. 

### 3.4. PCA Analysis of Cuprotosis-Related Genes, Cuprotosis-Related lncRNAs, and CRL Model lncRNAs

**Figure 5:**
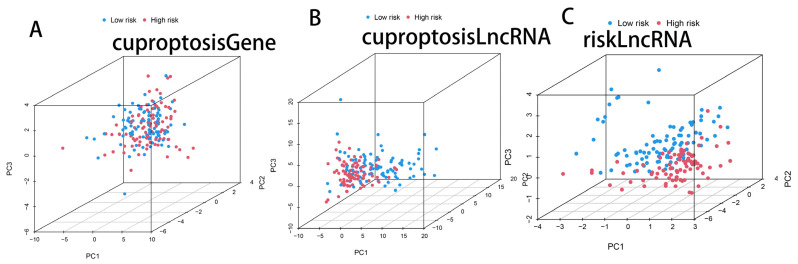
The CRL signature can better distinguish between high- and low-risk group patients. (**A**) PCA maps of the genes involved in cuprotosis. (**B**) lncRNAs associated with cuprotosis plotted using PCA. (**C**) lncRNA PCA plots using the signature of CRLs.

A PCA comparing cuprotosis-related lncRNAs, cuprotosis-related genes, and CRL model lncRNAs showed that the CRL model’s lncRNAs were more effective in classifying patients into high- and low-risk groups (Figure 5A–C). This demonstrates the efficacy of our method in differentiating between low- and high-risk populations.

### 3.5. Correlation Analysis between CRLs and Clinicopathological Features

**Figure 6:**
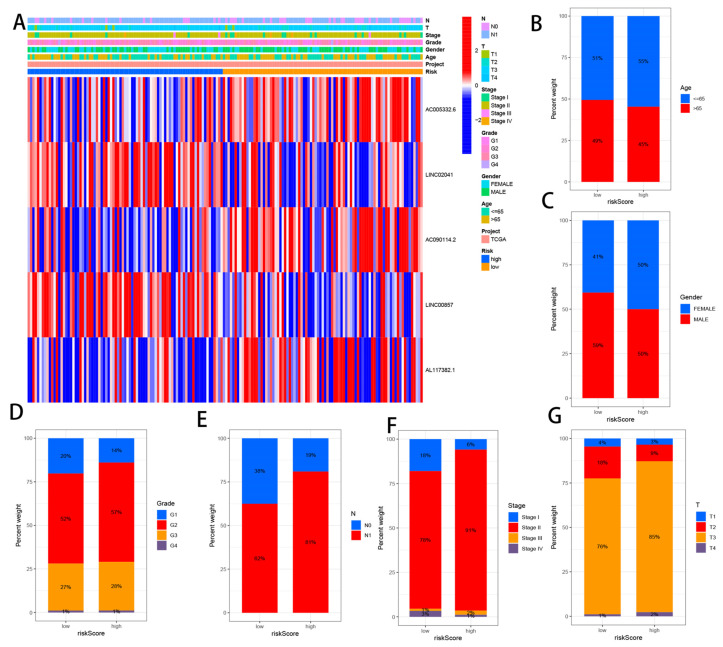
Correlation analysis between CRLs and clinicopathological features. (**A**) Heat map depicting the connection between the risk score and the clinicopathological traits. Between high-risk and low-risk groups, there were differences in the number of patients with different clinicopathological characteristics, including (**B**) age, (**C**) gender, (**D**) grade, (**E**) N stage, (**F**) stage, and (**G**) T stage.

The heat map shows the gender, age, grade, stage, T stage, N stage, and risk score of all TCGA head and neck squamous cell carcinoma patient samples (Figure 6A). We also found that the five CRLs significantly affected the frequency of certain clinicopathological features in the high-risk and low-risk groups (Figure 6B–G).

### 3.6. Clinical Subgroup Analysis of Risk Models for CRLs

**Figure 7:**
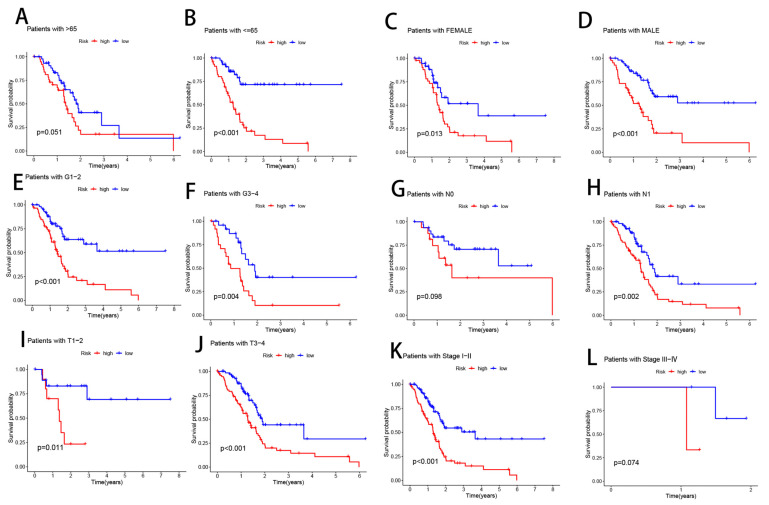
Prognostic power of the CRL risk model for overall survival for multiple PAAD subtypes: (**A**) age > 65 years; (**B**) age ≤ 65 years; (**C**) female; (**D**) male; (**E**) grades I–II; (**F**) grades III–IV; (**G**) N0; (**H**) N1; (**I**) T1–2; (**J**) T3–4; (**K**) stages I–III; (**L**) stages III–IV.

We compiled clinical data from the complete TCGA population to further explore whether there were changes in the prognosis of patients in various clinical groupings. Samples were then separated by age (>65 vs. ≤65), gender (male vs. female), tumor grade (I, II, III, IV), N stage (N0, N1), pathological stage (I, II, III, IV), and T stage (T1–2, T3, T4) to assess survival rates across these subgroups (Figure 7). Overall survival was substantially worse for high-risk patients compared to low-risk patients in all categories except those with ages >65, N0, and stages III–IV (Figure 4). These findings imply that the prognosis of various clinical subgroups of PAAD may be accurately predicted using our risk model for CRLs.

### 3.7. Combining Clinical Characteristics to Build Nomograms

**Figure 8:**
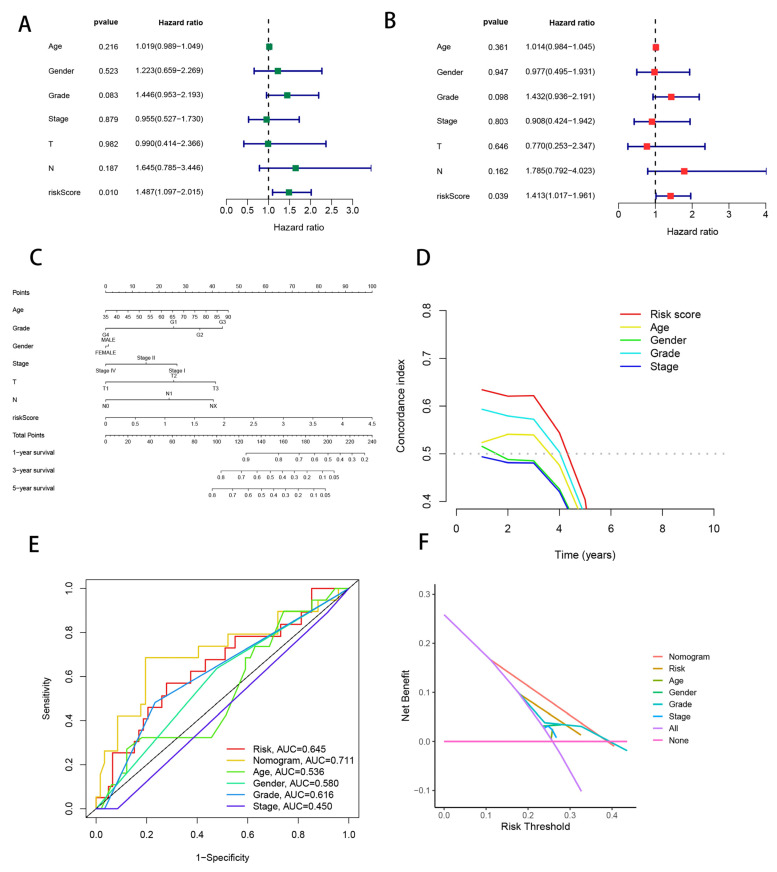
Independent prognostic analysis of CRL risk scores and clinical parameters. Univariate (**A**) and multivariate (**B**) Cox regression analysis of characteristics and different clinical features. (**C**) Nomogram for predicting 1-year, 3-year, and 5-year OS in PAAD patients. (**D**) C-index curves for risk scores and clinical parameters. (**E**) Multi-indicator ROC analysis of the test cohort. (**F**) Decision curve analysis. Green squares: univariate cox analysis of HR values for each variable. Red squares: multivariate cox analysis of HR values for each variable.

Given the considerable correlation between the risk model of CRLs and PAAD patient outcomes using univariate and multivariate Cox analyses, we wanted to see whether the five CRLs’ prognostic qualities might be employed as an independent factor in prognosis. The risk score was the only significant predictor of prognosis (*p* = 0.010) in the univariate analysis (Figure 8A). Further multivariate Cox analysis confirmed the risk score’s strong independent prognostic value (Figure 8B). We produced nomograms that employed these factors plus gender, age, stage, T stage, N stage, grade, and risk score to predict prognosis survival at 1, 3, and 5 years to improve the clinical usage and application of our risk model for PAAD’s five CRLs. The risk score was the most significant component in predicting OS, and the risk model based on the five CRLs’ genes offered the most accurate PAAD prediction (Figure 8C). Traditional clinicopathological characteristics were inferior to the CRL risk score model (AUC = 0.645) for predicting the prognosis of PAAD (Figure 8D,E). Our CRL risk model had the largest net benefit, which is consistent with this finding and suggests it has a greater impact on clinical decision-making (Figure 8F).

### 3.8. Enrichment Analysis of PAAD Patients Based on Prognostic Markers

**Figure 9:**
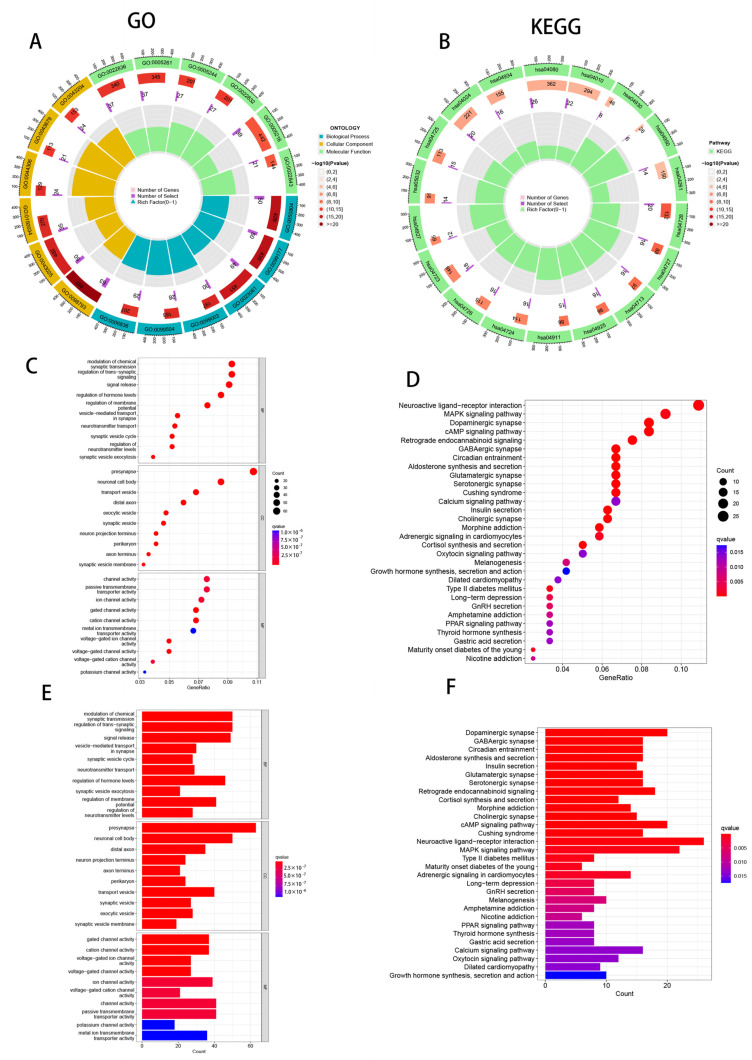
Gene Ontology (GO) and Kyoto Encyclopedia of Genes and Genomes (KEGG) pathway enrichment analysis. (**A**) Loop diagram showing apical GO signaling pathways involved in biological processes, cellular components, and molecular functions. (**B**) Loop diagram showing the apical KEGG signaling pathway. (**C**) Bubble plots of the first 10 GO-enriched words. (**D**) Bubble plots of the top 30 KEGG-enriched words. (**E**) Histogram of the top 10 GO-enriched words. (**F**) Histograms of the top 30 KEGG-enriched words.

The association between risk scores and bioactivity and signaling pathways was elucidated by means of KEGG enrichment analysis and GO functional analysis. We chose substantially enhanced items at FDR < 0.25 and p.adj < 0.05. These findings point to a strong relationship between CRLs and a variety of activities and processes, including those involved in the control of chemical synaptic transmission, the PPAR signaling system, and the MAPK signaling network (Figure 9).

### 3.9. Risk Score of CRLs Predicts Tumor Microenvironment and Immune Cell Infiltration

**Figure 10:**
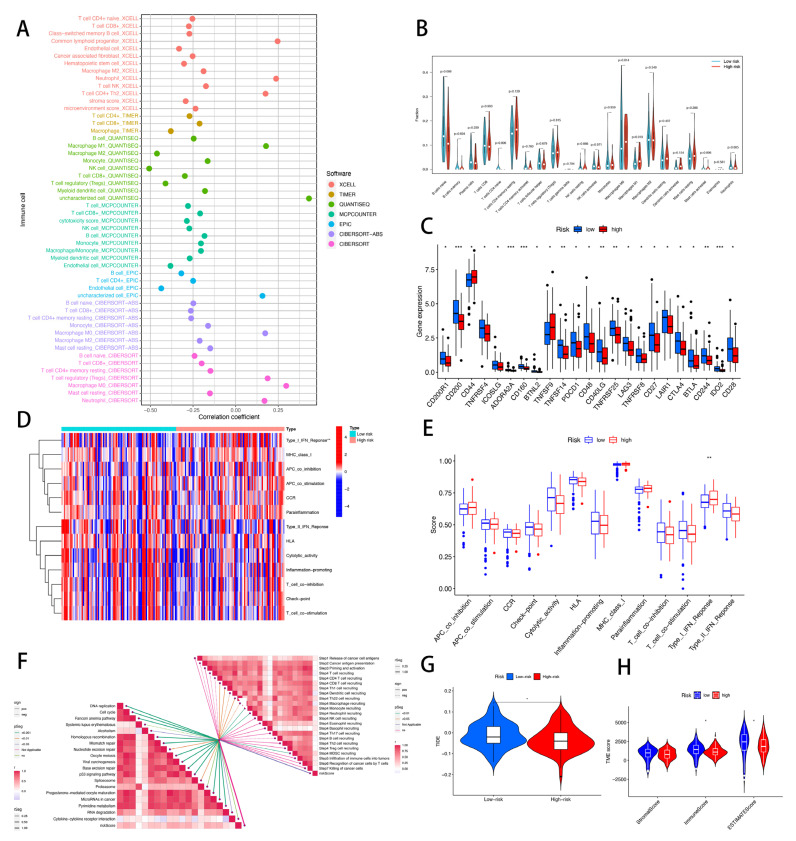
Risk scores for CRLs predict the tumor microenvironment and immunotherapy. (**A**) Immune cell bubble map. (**B**) Immune cell infiltration between high- and low-risk groups. (**C**) Immune checkpoint differences between high- and low-risk groups. (**D**,**E**) Differences in immune function between high- and low-risk groups. (**F**) Correlation of risk scores with ICB response signature and various steps of the tumor immune cycle. (**G**) Differences in TIDE scores between high- and low-risk groups. (**H**) Differences in TME scores between high- and low-risk groups. * *p* <0.05; ** *p* < 0.01; *** *p* < 0.001.

The tumor microenvironment (TME) immunological factors include immune checkpoint inhibitor (ICI) expression, tumor-infiltrating immune cell (TIIC) infiltration, and cancer-immunity cycle activity [38]. We first examined the risk score using the XCELL, TIMER, QUANTISEQ, MCPCOUNTER, CIBERSORT, CIBERSORT-ABS, and EPIC algorithms and searched for a link between the risk score and the quantity of invading immune cells (Figure 10A). Next, we used CIBERSORT to compare immune infiltration in the high- and low-risk groups. The only significant difference between the low-risk and high-risk groups was macrophage marker M1 expression (Figure 10B). Future investigations are needed to confirm this hypothesis; however, it seems that CRLs may particularly impact macrophage M1 expression and immunological activity, which in turn influences the response to immunotherapy in PAAD patients. Further changes in immune checkpoint expression were detected between the two groups, which is particularly noteworthy considering the significance of checkpoint-based immunotherapy. Interestingly, only two immune checkpoint genes, CD44 and TNFSF9, were significantly downregulated in the low-risk group, while twenty-one were significantly upregulated. These genes included CD200R1, CD200, TNFRSF4, ICOSLG, ADORA2A, CD160, BTNL2, TNSF14, PDCD1, CD48, CD40LG, TNFRSF25, LAG3, TNFRSF8, CD27, LAIR1, CTLA4, and BTLA (Figure 10C). In PAAD, an increased expression of CD44, a marker for cancer stem cells, was closely linked to tumor recurrence and a poor prognosis [39]. The above-mentioned discrepancies in the expression of invading immune cells between the high- and low-risk groups may be explained by the fact that TNFSF9 was strongly expressed in pancreatic cancer tissues and linked with the M1 polarization of macrophages [40]. As we had hypothesized, those with low CD44 and TNFSF9 expression were in the low-risk category, which had a better prognosis. Using risk scores, doctors may be able to adjust patients’ immunotherapy programs based on their unique expression of immune checkpoint genes. The immune function of PAAD patients was further investigated since invading immune cells and immune checkpoint genes play a significant role in immune function. Type I interferon response activity was found to be considerably lower in the low-risk group compared to the high-risk group (Figure 10D,E). We examined the relationship between risk scores and immunotherapy-predicted pathway enrichment scores since immunotherapy plays such a critical role in PAAD. The majority of the immune pathways were found to have a positive correlation with the risk score. These included DNA replication, progesterone-mediated oocyte maturation, cell cycle, alcoholism, homologous recombination, base excision repair, nucleotide excision repair, viral carcinogenesis, oocyte meiosis, the p53 signaling pathway, mismatch repair, the Fanconi anemia pathway, the spliceosome, microRNAs in cancer, pyrimidine metabolism, and RNA degradation (Figure 10F). We also studied the relationship between risk scores and the different stages of the tumor immune cycle to provide a more all-encompassing picture of tumor immunity. The findings did not have a statistically significant relationship with any of the stages of the tumor immune cycle. The results also showed a significant positive correlation of the risk score with the recruitment of CD8 T-cells, Th1 cells, Th22 cells, monocytes, neutrophils, and MDSCs (stage 4) and a significant negative correlation with the recruitment of DC cells and macrophages (stage 4) (Figure 10F), which may indicate that PAAD is a cold tumor. We also estimated the immunotherapy risk model’s likelihood using the tumor immunosuppression and rejection (TIDE) algorithm. Figure 10G shows that the TIDE score was substantially higher (*p* < 0.05) in the low-risk group than in the high-risk group, indicating that immune checkpoint inhibitor ICI) medication is less likely to help low-risk patients owing to their increased chance of immune evasion. Finally, we examined the tumor immune microenvironment and discovered that low-risk patients had a higher immunological score and ESTIMATE score than high-risk persons, suggesting a greater immune level and immunogenicity of the tumor microenvironment (Figure 10H).

### 3.10. Drug Sensitivity Analysis Related to CRLs

**Figure 11:**
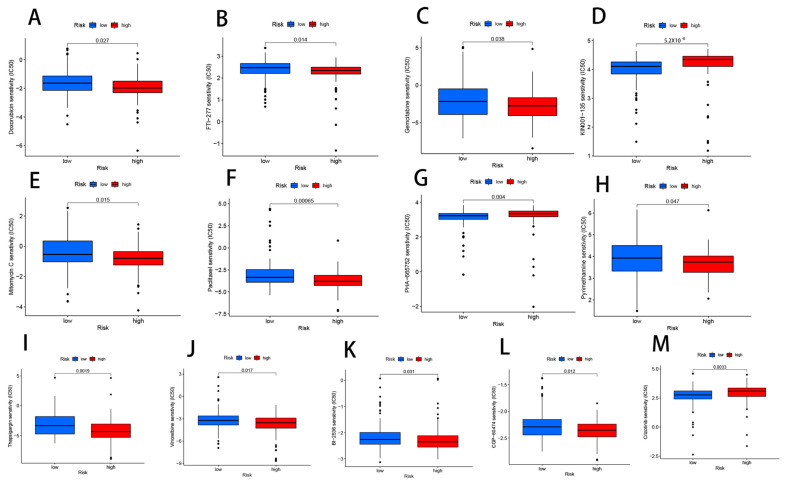
Differences in IC50 of immunotherapy drugs by risk score: (**A**) doxorubicin, (**B**) FTI-277, (**C**) gemcitabine, (**D**) KIN001-135, (**E**) mitomycin C, (**F**) paclitaxel, (**G**) PHA-665752, (**H**) pyrimethamine, (**I**) thapsigargin, (**J**) vinorelbine, (**K**) BI-2536, (**L**) CGP-60474, and (**M**) crizotinib.

Doxorubicin (*p* = 0.027), FTI-277 (*p* = 0.014), gemcitabine (*p* = 0.038), mitomycin C (*p* = 0.015), paclitaxel (*p* = 0.00065), pyrimethamine (*p* = 0.047), thapsigargin (*p* = 0.0019), vinorelbine (*p* = 0.017), BI-2536 (*p* = 0.031), and CGP-60474 (*p* = 0.012) were among the 13 immunotherapeutics. We also discovered that the IC50 for three additional chemical or targeted medicines was lower in the low-risk group: KIN001-135 (*p* = 5.2 × 10^−6^), PHA-665752 (*p* = 0.004), and crizotinib (*p* = 0.0033) (Figure 11A–M). The risk score allows for more in-depth analysis of an immunotherapy’s efficacy in treating PAAD patients and finer tuning of medication dosing.

### 3.11. Comparison of Somatic Mutations between Low- and High-Risk Groups

**Figure 12:**
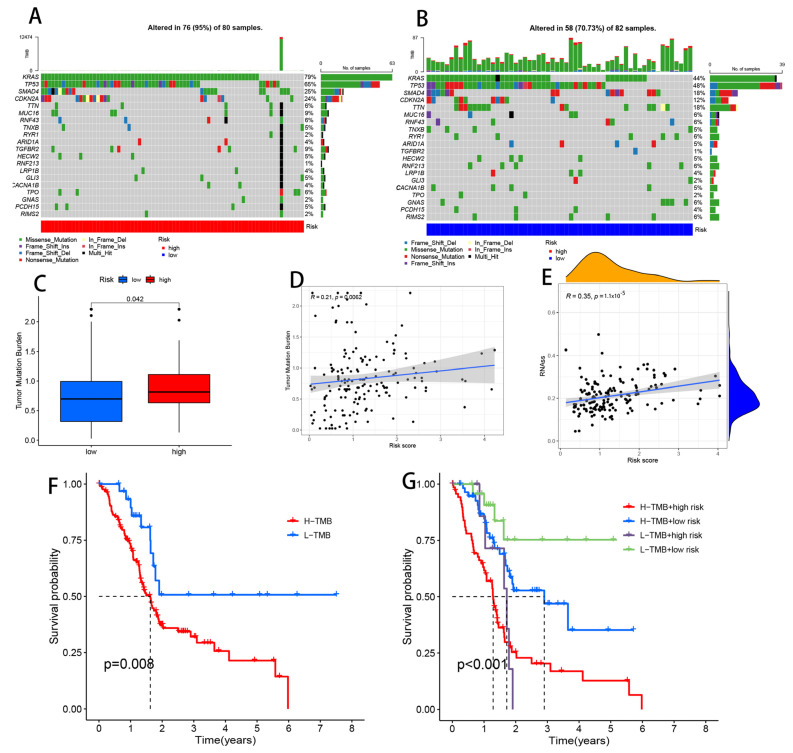
Landscape of mutation profiles in PAAD samples. (**A**,**B**) Pivotal mutation markers in the two groups. (**C**) TMB between high-risk and low-risk patients. (**D**) Correlation between TMB and risk score. (**E**) Correlation between risk score and CSC index. (**F**) Kaplan–Meier analysis shows the relationship between the level of TMB and clinical outcomes. (**G**) Effect of TMB with different risks on the probability of survival.

We divided PAAD patients into high- and low-risk categories and examined their somatic mutation data. KRAS (79%), TP53 (65%), and SMAD4 (25%) were the top three mutated genes in high-risk individuals. In contrast, the top four mutated genes in low-risk patients were KRAS (44%), TP53 (48%), SMAD4 (18%), and TTN (18%) (Figure 12A,B). Deletion of SMAD4 would eliminate the canonical TGF-β/SMAD4 signaling pathway and may make pancreatic cancer more aggressive [41]. Another study showed that patients with metastatic pancreatic cancer with a SMAD4 mutation had significant efficacy with a combination strategy consisting of chemotherapy (S-1) and cintilizumab [41]. Using somatic mutation data, we determined the TMB for both groups and found that it was considerably lower in the low-risk group as compared to the high-risk group (*p* = 0.042) (Figure 12C). We found a positive link between risk ratings and the TMB (*p* = 0.0062) in a correlation analysis (Figure 12D). We then examined the TMB as a PAAD prognostic biomarker. The median TMB value of each sample was used to split patients into two groups. The low-TMB group had better survival than the high-TMB group (*p* = 0.008) (Figure 12F). High-risk people had a high TMB, while low-risk people had both a low and a high TMB. Patients with a low risk and low TMB had the best prognosis, while patients with a high risk and high TMB had the worst (*p* < 0.001). PAAD patients’ prognoses can be predicted by the TMB and risk score (Figure 12G). In addition, we found that the TMB predicted the prognosis of PAAD patients in contrast to other common cancers, which was also confirmed by other studies [42], presumably because pancreatic cancer is a tumor with low immunogenicity [43], the specific reason for which could be the target of our next exploratory study.

Additionally, we investigated the possible connection between CRLs and pancreatic CSCs by analyzing mRNA expression (RNA). CRLs and CSC indices were shown to have a linear relationship (using RNA expression data; see Figure 12E) (RNAs). Overall, we found a significant correlation between CRLs and the CSC index (R = 0.35, *p* = 1.1 × 10^−5^), suggesting that greater CRLs are linked to more prominent stem cell characteristics and less cell differentiation.

### 3.12. qRT-PCR Assay in Cuprotosis Cell Model

**Figure 13:**
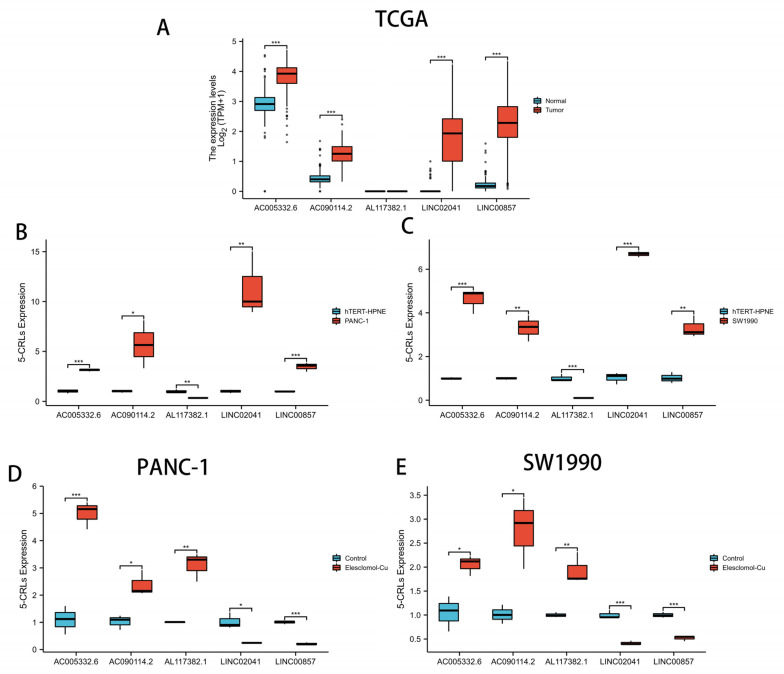
qRT-PCR assay in cuprotosis cell model. (**A**) The Wilcoxon rank-sum test was used to analyze the differential expression of 5 CRLs in PAAD tissues and normal tissues. (**B**,**C**) Expression levels of 5 CRLs in normal pancreatic cells and pancreatic cancer cells according to qRT-PCR. (**D**,**E**) qRT-PCR assay of 5 CRLs’ expression levels in the constructed cuprotosis cell model. * p < 0.05; ** p < 0.01; *** p < 0.001; ns > 0.05.

Differential expression analysis of the five CRLs using the “limma” package revealed that the expression levels of AC005332.6, AC090114.2, LINC00857, and LINC02041 were all upregulated in tumor tissues (*p* < 0.05), while the expression of AL117382.1 was not detected (Figure 13A). To validate the above results, we assessed the expression of the five CRLs at the transcriptional level using qRT-PCR and examined human PANC-1, SW1990, and hTERT-HPNE cells. The results showed that AC005332.6, AC090114.2, LINC00857, and LINC02041 were all upregulated in tumor cells, while AL117382.1 was downregulated in tumor cells (*p* < 0.05) (Figure 13B,C). In addition, to investigate whether the five CRLs are involved in the copper death process, we established a copper death model. The results showed that AC005332.6, AC090114.2, and AL117382.1 were significantly upregulated after drug action; LINC00857 and LINC02041 were significantly downregulated after drug action (Figure 13D,E). Therefore, we speculate that all five CRLs are likely to be involved in the copper death process of PAAD.

## 4. Discussion

PAAD is a prevalent type of malignancy in the digestive tract, although it is notoriously difficult to identify and cure [44]. More than half of pancreatic cancer patients are already in the metastatic stage when the cancer is discovered; thus, even though traditional therapies have improved, the prognosis is still bad [45]. Patients with a poor prognosis for PAAD require more investigation into useful and promising biomarkers and therapy targets. As with the use of any single component to predict a patient’s prognosis, the use of a single gene to predict PAAD prognosis has its caveats. Compared to a single biomarker, combinatorial models constructed from multiple related genes are more accurate in predicting prognosis and have important implications for the individualized treatment of tumor patients [46,47]. Recently, Tsvetko et al. proposed cuprotosis, a new mode of cell death, suggesting great potential for application in tumor therapy [9]. The proliferation rate of cancer cells can be regulated by cytotoxicity induced by the dynamic balance of copper [48]. In addition, cuprotosis can overcome the resistance of cancer cells to chemotherapy and help us to kill cancer cells effectively and selectively in immunotherapy [49]. Malignant characteristics such as tumor growth, invasion, and metastasis have been found to include lncRNAs in a number of investigations [50,51,52]. The outcome for those with PAAD improves drastically as a result of this. Nonetheless, CRLs’ potential prognostic significance in PAAD has not been investigated. Therefore, studying PAAD with CRLs can help to reveal the mechanism of PAAD’s development, which is crucial for the early detection and risk stratification of PAAD, further improving the survival of patients.

We developed a signature of five CRLs to define the prognostic prediction and immunological landscape of PAAD patients in order to better understand the possible role that CRLs may play in this disease. We used Lasso regression analysis and Cox risk regression analysis to identify five CRLs (AC005332.6, AC090114.2, LINC00857, LINC02041, and AL117382.1) that were shown to be independent prognostic variables for PAAD and correctly classified PAAD patients into two separate prognosis categories. Evidence suggests that AC005332.6, AC090114.2, and LINC02041 may serve as prognostic markers for many cancers, including pancreatic cancer [42,53,54,55,56]. Through a ceRNA-mediated mechanism, LINC00857 promotes the growth and invasion of pancreatic cancer cells [51]. Furthermore, LINC00857 regulates the miR-130b/RHOA axis [57], which in turn enhances the malignant characteristics of pancreatic cancer. Our findings that LINC00857 is substantially expressed and PAAD patients have a worse prognosis are supported by the aforementioned findings. By analyzing the ROC curve and the calibration curve, we were able to show that the signature of the five CRLs had a high degree of accuracy for making predictions. We developed a nomogram plot using clinical parameters and risk ratings to increase the prediction potential of the five CRLs’ signature and to show its practical utility in determining the prognosis of PAAD patients. The five CRLs were also investigated for their possible link to immune infiltration, immunotherapy success, medication responsiveness, and mutation in individuals with PAAD. Finally, we verified the possible involvement of the five CRLs in the cuprotosis process by constructing a cuprotosis model. Hence, the five-CRL signature can provide personalized immunotherapy and targeted therapy for PAAD patients and improve patient prognosis.

The TME is representative of a complex and dynamic environment of cellular and cell-free components with synergistic responses and functions in cancer progression [58,59]. As an important part of the TME, the immune microenvironment consisting of various immune cells is widely considered to be an important biomarker of tumors and a predictor of immune efficacy [60]. Tumor microenvironment (TME) immunology is crucial for several reasons, including ICI expression levels, TIIC infiltration, and the operation of the cancer immune circuit [38]. When we compared immune cell infiltration between the high- and low-risk groups, we found only changes in macrophage M1. Additionally, macrophage M1 was evaluated as a separate prognostic factor in PAAD [61]. 

As the most prominent immune cell of the TME, macrophages can promote tumor cell escape into the circulatory system and can suppress anti-tumor immune mechanisms and responses [62,63]. We investigated pancreatic cancer further and found that pancreatic cancer stimulates inflammatory M1 macrophages to produce factors including NF-B and Notch, which in turn leads to carcinogenesis [64,65]. Additionally, we observed that TNFSF9 is substantially expressed in individuals with high-risk PAAD and that TNFSF9 is related to the M1 polarization of macrophages [40] while refining another major immunological characteristic of the TME, immune checkpoint inhibitors (ICIs). Activation of Src/FAK/p-Akt/IL-1β signaling by TNFSF9 is shown to increase pancreatic cancer metastasis by modulating the M2 polarization of macrophages [66]. We speculate that TNFSF9’s regulation of macrophage M1 may have a similar mechanism and could be the next step in the study. In summary, there may be multiple mechanisms that contribute to the increase in macrophage M1 in pancreatic cancer patients in the high-risk group. CD44 is also highly expressed in high-risk groups. The role of CD44 has been extensively studied in various tumors, such as intestinal cancer [67,68], breast cancer [69], and ovarian cancer [70], and is closely related to chemotherapeutic drug sensitivity and tumor cell adhesion and migration [70], but there is still a large gap in PAAD. There are also many immune checkpoints that are upregulated in expression in patients in the low-risk group. The expression of CD200 in the pancreatic cancer microenvironment can amplify MDSCs, and targeting CD200 can enhance the activity of checkpoint immunotherapy [71]. Patients in the low-risk category of pancreatic cancer may have comparable resistance mechanisms to those seen in breast cancer, where ICOSLG has been shown to be a possible biomarker for the development of trastuzumab resistance [72]. New clinical evidence shows that BTNL2 expression is elevated following anti-PD-1 treatment, lending credence to the concept that it may play a crucial role in a novel mechanism of cancer immune escape [73]. An increase in CTLA4 and PD-1 expression was seen in the low-risk group, and anti-PD-1/PD-L1 antibodies were less effective against pancreatic cancer than they were against melanoma, renal cell carcinoma, and non-small cell lung cancer, owing to immunostasis or drug resistance [74,75,76]. LAG-3-expressing tumor-infiltrating T-cells were associated with a worse chance of progression-free survival in patients with pancreatic cancer [77,78]. This may be because LAG-3 expression inhibits CD4+ T-cell activation, increases Treg suppressive activity, and decreases cytotoxic CD8+ T-cell function. When it comes to pancreatic cancer, IDO2 is being looked at as a potential new biological target. Pancreatic cancer cells have been shown to upregulate IDO2 as a means of achieving immunological tolerance via localized changes in the immune environment [79]. It is essential to increase the response to immune checkpoint blockage in PAAD patients by combining other medications to transform pancreatic cancer into a hot tumor [80]. Targeted blockade of IL-6 combined with checkpoint blockade has been found to increase the number of infiltrating T lymphocytes in pancreatic cancer tumors, resulting in better outcomes [81].

It is also important to recognize the role played by the cancer immunity cycle in shaping the immunological microenvironment of tumors. The so-called “cancer-immunity cycle” is dysfunctional in people with cancer. The lack of the detection of tumor antigens and DCs and T-cells mistaking self-antigens for foreign ones might lead to a T regulatory cell response rather than an effector cell response [82]. Comprehensively reflecting the ultimate result of the intricate immunomodulatory interactions in the TME is the dynamic cancer immunity cycle [83]. As a result, we investigated how cancer risk scores correlated with the different checkpoints in the vaccination process. In order to better assess the result of immune control of the tumor microenvironment, we may utilize the risk score to forecast the execution of each phase of the cancer immunity cycle in patients.

Mutations are processed into neoantigens and presented to T-cells by major histocompatibility complex (MHC) proteins. The higher the TMB, the more likely it is to produce immunogenic neoantigens that can trigger a T-cell response [84]. Cancer suppresses immune checkpoints and creates immune escape, while immune checkpoint inhibitors (ICIs) alter cancer therapy by reactivating T-cells [84]. Clinical investigations on a wide variety of tumor types have shown a significant correlation between PD-1/PD-L1 inhibitor effectiveness and the TMB in PD-L1-positive populations, indicating that the TMB may be a suitable biomarker for predicting PD-1/PD-L1 drug efficacy [84,85]. This great prospect of application and effect is exciting, but also faces many issues, which need to be urgently addressed. PD-L1 expression and the TMB are not significantly correlated in most cancer subtypes [86,87,88]. Additionally, the TMB may not necessarily correspond to ICI responsiveness [84]. As a consequence, evaluating patients’ ICI response requires correlating the TMB with other responsiveness/resistance biomarkers, which may provide more accurate findings. The results of this research showed a strong association between CRLs and the TMB, suggesting that CRL levels may serve as an indirect indicator of TMB status and, as such, be of great use in predicting the success of immunotherapy. Higher TMB levels in the high-risk group were seen, which is consistent with a greater potential for immunotherapy success in that subset. To our surprise, we discovered that an elevated TMB is closely linked to a poor prognosis of PAAD, because pancreatic cancer is an immune-evasive tumor [43]. The TMB and risk ratings worked well together to forecast PAAD patients’ outcomes. This means that our results may be used as a standard and as a basis for clinical practice to direct immunotherapy in PAAD.

Though our work has substantial clinical significance for determining prognosis and selecting treatments for individuals with PAAD, it does have significant limitations. First, individual differences in PAAD patients may affect the five CRLs’ signature, and this was only assessed on the TCGA dataset. Although we tried to obtain external datasets (GEO and ICGC) for validation, we were still unable to obtain correct lncRNA information due to the bias and limitations of the microarray data. Second, although we were able to build a cuprotosis model and find evidence that five CRLs may be involved in the cuprotosis process of PAAD using qRT-PCR, the mechanism behind this association has to be investigated further through in vivo and in vitro research.

## 5. Conclusions

In summary, we identified cuprotosis programmed-cell-death-related lncRNAs as a novel prognostic biomarker and potential therapeutic target for PAAD patients. In addition, we successfully constructed a prognostic signature consisting of five CRLs that can accurately assess the prognosis of PAAD patients and characterize the immune landscape of PAAD patients, helping clinicians to identify specific subgroups of patients who may benefit from immunotherapy and chemotherapy for personalized treatment.

## Data Availability

The datasets analyzed in the current study are available in the TCGA repository (http://cancergenome.nih.gov/) (accessed on 2 August 2022). The datasets used and/or analyzed during the current study are available from the corresponding author upon reasonable request.

## Supplementary Materials

The following Supporting Information can be downloaded at: https://www.mdpi.com/article/10.3390/cells11213436/s1, Table S1: The primer sequences for qPCR assay.
