# Supplementary material for: Cuprotosis Programmed-Cell-Death-Related lncRNA Signature Predicts Prognosis and Immune Landscape in PAAD Patients
Source: Cells. 2022 Oct 31;11(21):3436. doi: 10.3390/cells11213436 (PMC9658590; doi:10.3390/cells11213436)
Supplement: Supplementary file 1 [file cells-11-03436-s001.zip › cells-1925672-supplementary.pdf]

Supplementary Table S1. The primer sequences for qPCR assay.

Gene	Forward primer	Reverse primer
**GAPDH**	TGACATCAAGAAGGTGGTGAAGCAG	GTGTCGCTGTTGAAGTCAGAGGAG
**AC005332.6**	ATCGCATGTGTCCTGTCTTCTGAAC	CCACTCGTGAGCACTGACAACTG
**AC090114.2**	GTTCCACTGCCTTCTCCACACTTC	GGTGGTGATGGTCGTGCTGTTC
**AL117382.1**	GGCAACCTGAAGAGATGTGGAGAC	ATACTGCTGATGCGGACCAATGC
**LINC02041**	GGAGCCCAGCCAAGTAATGAAGC	AATGAATCAGGACAGCCGTCAAG TG
LINC00857	GCCTCCGTTAAGCACCAGAAGTC	ATTGGGACAGGGTTTGGAACTCTT G
